# ﻿Several new combinations from previous *Didymocarpus* to *Palmatiboea* (Gesneriaceae)

**DOI:** 10.3897/phytokeys.250.138694

**Published:** 2024-12-09

**Authors:** Fang Wen, Qiang Fan

**Affiliations:** 1 Guangxi Key Laboratory of Plant Conservation and Restoration Ecology in Karst & Terrain, Guangxi Institute of Botany, Guangxi Zhuang Autonomous Region and Chinese Academy of Science, Guilin 541006, China; 2 Gesneriad Committee of China Wild Plant Conservation Association (GC), National Gesneriaceae Germplasm Resources Bank of GXIB (NGGRB), Gesneriad Conservation Center of China (GCCC), Guilin 541006, Guangxi, China; 3 State Key Laboratory of Biocontrol and Guangdong Provincial Key Laboratory of Plant Stress Biology, School of Life Sciences, Sun Yat-sen University, Guangzhou 510275, China; 4 National Park and Nature Education Research Institute, Sun Yat-sen University, Guangzhou 510275, China

**Keywords:** China, *Didymocarpus*, *Didymocarpuspingyuanensis*, Gesneriacese, nomenclature, *Palmatiboea*

## Abstract

The recently published species, *Didymocarpuspingyuanensis*, is transferred here to the recently re-circumscribed genus, *Palmatiboea*. Two varieties, recently assigned to *Palmatiboea*, have been raised to the rank of species. Their Chinese vernacular names are also revised and provided here.

## ﻿Introduction

The genus, *Didymocarpus* Wall., which was established over 200 years ago (Wallich 1819), has become one of the most frequently revised genera within Gesneriaceae. Because of different taxonomic perspectives, morphological evidence has revealed significant macroscopic and even microscopic variation both within and between species of the genus, with highly diverse geographical distributions further complicating its classification. As a result, the genus has gained a reputation as a “repository” or “dumping ground” for morphologically disparate species. At the same time, it has also incorporated a considerable number of species that are highly similar in external morphology (Burtt and Wiehler 1995; Burtt 1997; Vitek et al. 2000; Weber et al. 2000; Liu et al. 2024).

A revision 27 years ago defined the narrow sense of *Didymocarpus* s. str., which includes two sections: Didymocarpussect.Didymocarpus and Didymocarpussect.Elati Ridl., distributed across Southwest China, the greater Himalayan region, and the Indochinese Peninsula (Weber and Burtt 1997). However, the Chinese species of this genus were divided into two sections: stemmed herbaceous plants (Didymocarpussect.Didymocarpus) and stemless herbaceous plants (Didymocarpussect.Heteroboea W.T.Wang) (Wang et al. 1990, 1998; Li and Wang 2005). *Sect. Heteroboea* was originally defined based on morphological characteristics, but recent systematic studies and morphological comparisons have reassigned five species to the genus *Petrocodon* Hance. These species are: *P.bonii* (Pellegr.) A.Weber & Mich.Möller, distributed along the northern edge of the Indochina Peninsula; *P.mollifolius* (W.T.Wang) A.Weber & Mich.Möller, *P.subpalmatinervis* (W.T.Wang) F.Wen & Z.L.Li, and *P.niveolanosus* (D.Fang & W.T.Wang) A.Weber & Mich.Möller, found in Southwest China; and *P.hancei* (Hemsl.) Mich.Möller & A.Weber, distributed in South China (Guangxi and Guangdong) and Central China (Hunan) (Weber et al. 2011; Li et al. 2023).

After the publication of *Flora Reipublicae Popularis Sinicae* (Vol. 69) (Wang et al. 1990), four new taxa were discovered and described within sect. Heteroboea: *Didymocarpusdissectus* F.Wen, Y.L.Qiu, Jie Huang & Y.G.Wei (Wen et al. 2013), found in Fujian Province; D.heucherifoliusHand.-Mazzvar.yinzhengii J.M.Li & S.J.Li (Li and Li 2014), found in Hunan Province; D.heucherifoliusHand.-Mazzvar.gamosepalus Xin Hong & F.Wen (Xu et al. 2019), found in Guangdong Province; and *D.lobulatus* F.Wen, Xin Hong & W.Y.Xie (Xie et al. 2020), found in Zhejiang Province.

After multiple revisions, the number of species included in the genus *Didymocarpus* has decreased from over 180 species at its peak to approximately 110 species. However, some unresolved taxonomic issues remain. Recently, the study by Liu et al. (2024) addressed the longstanding taxonomic status of sect. Heteroboea, which is endemic to China and has been a subject of debate within *Didymocarpus* s. str.

Liu et al. (2024) conducted phylogenetic analyses with comprehensive sampling across *Didymocarpus* and related genera, utilizing four nuclear ribosomal DNA markers and five chloroplast DNA regions. The results revealed that *Didymocarpus* is not monophyletic. Based on a combination of molecular phylogenetic and morphological data, the circumscription of *Didymocarpus* s. str. was redefined. A new genus, *Palmatiboea* F.P.Liu & Yin Z.Wang, is established to accommodate those species previously assigned to Didymocarpussect.Heteroboea. *Palmatiboea* is clearly differentiated from *Didymocarpus* s. str., not only in molecular and morphological characteristics but also in its distinct geographic distribution, as it is restricted to Southeast and South China. Table 1 shows the species originally classified under *Didymocarpus* that have been revised and reassigned to the genus *Palmatiboea* by Liu et al. (2024).

**Table 1.** The species originally from *Didymocarpus* that have been reassigned to *Palmatiboea* in Liu et al. 2024.

No.	The original scientific names of species in *Didymocarpus*	The original Chinese name	The revised species scientific names in *Palmatiboea*	The revised Chinese name
1	*Didymocarpuscortusifolius* (Hance) Lévl.	温州长蒴苣苔	*Palmatiboeacortusifolia* (Hance) F.P.Liu & Y.Z.Wang*	温州掌脉苣苔
2	*Didymocarpusdissectus* F.Wen, Y.L.Qiu, Jie Huang & Y.G.Wei	深裂长蒴苣苔	*Palmatiboeadissecta* (F.Wen, Y.L.Qiu, Jie Huang & Y.G.Wei) F.P.Liu & Y.Z.Wang*	深裂掌脉苣苔
3	*Didymocarpusheucherifolius* Hand.-Mazz.	闽赣长蒴苣苔	*Palmatiboeaheucherifolia* (Hand.-Mazz.) F.P.Liu & Y.Z.Wang*	闽赣掌脉苣苔
4	Didymocarpusheucherifoliusvar.gamosepalus Xin Hong & F.Wen	合萼长蒴苣苔	Palmatiboeaheucherifoliavar.gamosepala (Xin Hong & F.Wen) F.P.Liu & Y.Z.Wang*	合萼掌脉苣苔
5	Didymocarpusheucherifoliusvar.yinzhengii J.M.Li & S.J.Li	印政长蒴苣苔	Palmatiboeaheucherifoliavar.yinzhengii (J.M.Li & S.J.Li) F.P.Liu & Y.Z.Wang*	印政掌脉苣苔
6	*Didymocarpuslobulatus* F.Wen, Xin Hong & W.Y.Xie	浙东长蒴苣苔	*Palmatiboealobulata* (F.Wen, Xin Hong & W.Y.Xie) F.P.Liu & Y.Z.Wang*	浙东掌脉苣苔
7	*Didymocarpussinoprimulinus* W.T.Wang	报春长蒴苣苔	*Palmatiboeasinoprimulia* (W.T.Wang) F.P.Liu & Y.Z.Wang*	报春掌脉苣苔
8	*Didymocarpusreniformis* W.T.Wang	肾叶长蒴苣苔	*Palmatiboeareniformis* (W.T.Wang) F.P.Liu & Y.Z.Wang*	肾叶掌脉苣苔
9	*Didymocarpus salviiﬂorus* Chun	迭裂长蒴苣苔	*Palmatiboea salviiﬂora* (Chun) F.P.Liu & Y.Z.Wang*	迭裂苣苔（应更正为迭裂掌脉苣苔） Should be changed as 迭裂掌脉苣苔
10	*Didymocarpusyuenlingensis* W.T.Wang	沅陵长蒴苣苔	*Palmatiboeayuenlingensis* (W.T.Wang) F.P.Liu & Y.Z.Wang*	沅陵掌脉苣苔

^*^: Inaccurate species namer’s abbreviation.

However, Liu et al. (2024) did not elevate the two infraspecific taxa (varieties) listed in Table 1 to species rank. In a nearly concurrent study on Didymocarpussect.Heteroboea distributed in southern to southeastern China, two varieties mentioned in Table 1 have already been elevated to the species level: Didymocarpusheucherifoliusvar.gamosepalus Xin Hong & F.Wen was revised to *D.gamosepalus* (Xin Hong & F.Wen) Ling H.Yang, Q.Fan & F.Wen, and D.heucherifoliusvar.yinzhengii J.M.Li & S.J.Li was revised to *D.yinzhengii* (J.M.Li & S.J.Li) Ling H.Yang, Q.Fan & F.Wen (Yang et al. 2024). The two research teams focused on different taxonomic levels—one at the genus level and the other at the species level—leading to slight discrepancies in their conclusions on the same taxonomic issues.

In the study by Yang et al. (2024), a new species, *Didymocarpuspingyuanensis* Ling H.Yang, Q.Fan & F.Wen, from northeastern Guangdong, China, was described and compared with D.heucherifoliusvar.gamosepalus and *D.salviiflorus*, two species that have since been transferred to the genus *Palmatiboea* as P.heucherifoliusvar.gamosepalus and *P.salviiflora*. Based on the morphological characteristics of this new species, *Didymocarpuspingyuanensis* aligns closely with the concept of *Palmatiboea*, suggesting that it should be transferred to the new genus.

## ﻿Nomenclature

### ﻿ Palmatiboea pingyuanensis

Taxon classificationPlantaeLamialesGesneriaceae

(Ling H.Yang, Q.Fan & F.Wen) F.Wen & Q.Fan comb. nov.

F7A967AA-1F8B-53B9-90B4-373044FD72BF

urn:lsid:ipni.org:names:77353132-1

≡ Didymocarpuspingyuanensis Ling H.Yang, Q.Fan & F.Wen in Yang et al., Phytokeys 224: 218 (2024).

#### Type.

China • Guangdong Province: Meizhou City, Pingyuan Town, 24°32'N, 115°50'E, 491 m a.s.l., 1 April 2023 (fl.), Qiang Fan, Xing-yue Zhang, Li-Juan Liao, Jie-Hao Jin, Ling-Han Yang DNPC 3352 (holotype: SYS!; isotypes: IBK! IBSC! SYS!)

#### The Chinese vernacular name.

平远掌脉苣苔 (Píng Yuǎn Zhǎng Mài Jù Tái).

### ﻿ Palmatiboea yinzhengii

Taxon classificationPlantaeLamialesGesneriaceae

(J.M.Li & S.J.Li.) F.Wen & Q.Fan comb. nov.

BE4D2458-A9A2-50E0-A145-0E0E98ADDC01

urn:lsid:ipni.org:names:77353133-1

≡ Didymocarpusheucherifoliusvar.yinzhengii J.M.Li & S.J.Li, in Phytotaxa 156 (3): 187. 2014.
≡ Palmatiboeaheucherifoliavar.yinzhengii (J.M.Li & S.J.Li) F.P.Liu & Y.Z.Wang, in Journal of Systematic and Evolution doi: 10.1111/jse.13124:13.2024.

#### Type.

China • Hunan: near Yongxing County. alt. 300 m, 26°17'10"N, 113°11'25"E, 6 May 2011, Jia-Mei Li 1105062 (holotype: HEAC!); ibid. Jia-Mei Li 11501 (paratype: IBK!).

### ﻿ Palmatiboea gamosepalus

Taxon classificationPlantaeLamialesGesneriaceae

(Xin Hong & F.Wen) F.Wen & Q.Fan comb. nov.

A4C6E988-719C-5E53-BB51-DBFB013336D6

urn:lsid:ipni.org:names:77353134-1

≡ Didymocarpusheucherifoliusvar.gamosepalus Xin Hong & F.Wen, in Phyto­Keys 128: 34. 2019.
≡ Palmatiboeaheucherifoliavar.gamosepala (Xin Hong & F.Wen) F.P.Liu & Y.Z.Wang, in Journal of Systematic and Evolution doi: 10.1111/jse.13124: 13. 2024.

#### Type.

China • Guangxi Province, cultivated in the nursery of Gesneriad Conservation Center of China (GCCC), introduced from north of Guangdong Province: Pingyuan County, Meizhou City, growing in rocky crevices at the foot of a calcareous sedimentary rocky hill. 22 February 2019, flowering, WF20190222-05 (holotype: IBK!; isotype: AHU!)

#### Notes.

We have noticed that several newly revised species and genera names use the name abbreviation of the corresponding author Yin-Zheng Wang as “Y.Z.Wang,” such as the names of the two new genera, *Palmatiboea* F.P.Liu & Y.Z.Wang and *Hequnia* Y.Z.Wang & F.P.Liu in Liu et al. (2024). However, the name abbreviation “Y.Z.Wang” originates from Prof. Wang Yun-Zhang, a renowned Chinese mycologist and plant pathologist, whose name abbreviation first appeared in the 1980s when he described 15 new species of Rust Fungi (Wang et al. 1980). Prof. Wang Yin-Zheng, on the other hand, first published a new taxon in the mid-1990s, *Whytockiapurpurascens* Yin Z.Wang and *W.hekouensis* Yin Z.Wang (Wang 1995). When *W.purpurascens* and *W.hekouensis* were published, the author’s name abbreviation was mistakenly written as “Y.Z.Wang.” However, the abbreviation “Y.Z.Wang” for Prof. Wang Yun-Zhang appeared 15 years earlier than that of Prof. Wang Yin-Zheng when describing new taxa (IPNI 2024); therefore, for the aforementioned nomenclature, including the new genera *Palmatiboea* and *Hequnia* and their respective species. Additionally, there are quite a few revised species of Gesneriaceae that incorrectly use “Y.Z.Wang” to refer to Prof. Wang Yin-Zheng. For example, PetrocosmeashilinensisY.M.Shui & H.T.Zhaovar.changhuensis T.F.Lü & Y.Z.Wang should be changed as P.shilinensisvar.changhuensis T.F.Lü & Yin Z.Wang (Li et al. 2020), and so on. In summary, the correct name abbreviation should be “Yin Z.Wang,” not “Y.Z.Wang” among the many revised species epithets of Gesneriaceae mentioned above.

## Supplementary Material

XML Treatment for Palmatiboea pingyuanensis

XML Treatment for Palmatiboea yinzhengii

XML Treatment for Palmatiboea gamosepalus

## ﻿Additional information

### Author ORCIDs

Fang Wen https://orcid.org/0000-0002-3889-8835

Qiang Fan https://orcid.org/0000-0003-4254-6936

### Data availability

All of the data that support the findings of this study are available in the main text.
